# Feeding of *Hermetia illucens* Larvae Meal Attenuates Hepatic Lipid Synthesis and Fatty Liver Development in Obese Zucker Rats

**DOI:** 10.3390/nu15020287

**Published:** 2023-01-06

**Authors:** Magdalena J. M. Marschall, Sarah M. Grundmann, Denise K. Gessner, Gaiping Wen, Erika Most, Klaus Eder, Robert Ringseis

**Affiliations:** Institute of Animal Nutrition and Nutrition Physiology, Justus Liebig University Giessen, 35392 Giessen, Germany

**Keywords:** *Hermetia illucens*, Zucker rat, lipid metabolism, fatty liver, insect protein, fatty acid synthesis, chitin

## Abstract

The present study tested the hypothesis that dietary insect meal from *Hermetia illucens* (HI) larvae attenuates the development of liver steatosis and hyperlipidemia in the obese Zucker rat. To test the hypothesis, a 4-week trial with male, obese Zucker rats (n = 30) and male, lean Zucker rats (n = 10) was performed. The obese rats were assigned to three obese groups (group O-C, group O-HI25, group O-HI50) of 10 rats each. The lean rats served as a lean control group (L-C). Group L-C and group O-C were fed a control diet with 20% casein as protein source, whereas 25% and 50% of the protein from casein was replaced with protein from HI larvae meal in the diets of group O-HI25 and O-HI50, respectively. The staining of liver sections with Oil red O revealed an excessive lipid accumulation in the liver of group O-C compared to group L-C, whereas liver lipid accumulation in group O-HI25 and O-HI50 was markedly reduced compared to group O-C. Hepatic concentrations of triglycerides, cholesterol, C14:0, C16:0, C16:1, C18:0, C18:1, the sum of total fatty acids and hepatic mRNA levels of several genes associated with lipid synthesis and plasma concentration of cholesterol were markedly higher in group O-C than in group L-C, but lower in group O-HI50 than in group O-C (*p* < 0.05). In conclusion, partial replacement of casein by HI larvae meal attenuates liver steatosis and dyslipidemia in obese Zucker rats. This suggests that HI larvae meal serves as a functional food protecting from obesity-induced metabolic disorders.

## 1. Introduction

One of the major challenges in the near future is to cover the growing demand for food of the rising world population despite the increasing scarcity of natural resources (e.g., arable land and water) required for agricultural production. At the same time, the environmental burden from agricultural production, such as greenhouse gas (GHG) emissions, must be reduced in order to reach the global climate protection goals and contribute towards carbon neutrality. Against this background, agricultural production must be intensified in a sustainable way, so that more food is produced with fewer resources and fewer emissions. In this regard, the bioconversion of organic agro-industrial side streams into edible protein-rich biomass by insects has been recognized as a suitable strategy to face this challenge [1,2]. This is attributed to the fact that large-scale mass production of insect biomass in a circular food system allows resource-efficient recycling of regionally available agro-industrial co- and by-products, and is associated with lower usage of natural resources (space, water, energy) and lower environmental impact (e.g. less GHG emissions) than required for the production of conventional protein sources [3,4]. Amongst several insect species approved by the European Commission for insect-based food and feed, two species, *Tenebrio molitor* (TM) and *Hermetia illucens* (HI), have emerged as the most promising for resource-efficient mass production of protein-rich insect biomass such as insect larvae meal [5,6].

Apart from serving as a source of essential amino acids, which are required as building blocks for protein synthesis in both humans and monogastric farm animals, insect larvae meal might be interesting especially in humans due to pleiotropic health-related effects. For instance, dietary TM larvae meal was shown to cause antiadipogenic effects in high fat diet (HFD)-induced obese mice and to inhibit lipogenesis in cultured adipocytes [7]. In addition, several studies demonstrated strong liver and plasma lipid-lowering effects and a pronounced inhibition of hepatic lipogenesis of partially defatted TM larvae meal in the obese Zucker rat [8,9,10], an established model of liver steatosis, obesity, diabetes and metabolic syndrome. Likewise, Lee et al. [11] reported that TM larvae meal decreases lipid accumulation and lipogenesis in the liver of HFD-induced obese mice. Although the bioactive compounds responsible for the inhibitory effect of TM larvae meal on lipogenesis remain to be identified, different constituents of TM larvae meal may be likely candidates for its lipid-lowering action. One is chitin, an intrinsic constituent of the insect’s exoskeleton, which makes up 9–13% of dry matter in TM larvae meal [8,12], and has been demonstrated to cause lipid-lowering effects [13]. Other possible candidates are biologically active peptide sequences, which have been found in different dietary proteins including milk protein [14], egg protein [15], soybean protein [16,17] and peanut protein [18]. Such bioactive peptides, which have been also identified in TM larvae protein [19,20], are known to be released during protein digestion and enter the circulation in intact form to mediate different biological effects, such as lipid-lowering or hypotensive activities. Since all of the abovementioned health-related effects have been observed with the use of TM larvae meal, the question arises of whether such effects can be also observed with the use of HI larvae meal, which also contains chitin (6.8% [21]) and presumably also bioactive peptides. This, however, is currently unknown from the available literature. In order to close this gap of knowledge, the hypothesis was tested that dietary HI larvae meal attenuates liver steatosis development and hyperlipidemia in the obese Zucker rat, which is an established rodent model of liver steatosis and hyperlipidemia.

## 2. Materials and Methods

### 2.1. Animals and Diets

The animal experiment was approved by the Animal Welfare Officer of the Justus Liebig University Giessen (approval no.: JLU 786_M). All experimental procedures described followed established guidelines for the care and handling of laboratory animals. The experiment included 30 male, 7–8-week-old, homozygous (fa/fa) obese Zucker rats (Crl:ZUC-Lepr^fa^) and 10 male, 7–8-week-old, heterozygous (fa/+) lean Zucker rats. The rats were obtained from Charles River (Sulzfeld, Germany). The animals were kept in groups of two animals each under controlled conditions (12-h light:12-h dark, 22 ± 1 °C ambient temperature, 50–60% relative humidity). The obese rats were randomly assigned to three groups of ten rats each [obese casein (O-C), obese with HI larvae meal at a casein replacement level of 25% (O-HI25), obese with HI larvae meal at a casein replacement level of 50% (O-HI50)]. The lean rats served as a control group without pathological disruptions in feed intake regulation and lipid metabolism (L-C) and were fed the same diet as group O-C. The composition of the three semisynthetic diets is shown in Table 1. Nutrient levels were sufficient to meet requirements of the rat for maintenance according to the National Research Council (NRC) [22]. The diet C, which was fed to groups L-C and O-C, contained casein as the only protein source, whereas 25% and 50% of the protein from casein [*N* × 6.25 = 915 g/kg fresh matter (FM)] was replaced with protein from HI larvae meal [*N* × 4.67 = 395 g/kg FM] in diets HI25 and HI50, respectively. While the protein content is usually calculated from total *N* using the *N*-to-protein conversion factor of 6.25, this factor overestimates the protein content due to the presence of nonprotein *N*, such as chitin, in insects. In order to avoid overestimation of the protein in HI larvae meal, its protein content was calculated using a HI larvae-specific *N*-to protein conversion factor of 4.67 [23]. The partially defatted HI larvae meal was provided from Madebymade GmbH (Pegau, Germany). The company´s details on the rearing conditions (substrate, duration) and further processing of the HI larvae are not available for reasons of confidentiality.

The analyzed concentrations of crude nutrients, chitin and fatty acids of the HI larvae meal are shown in Table 2. All diets contained 0.5% titanium dioxide (TiO_2_) as an indicator to calculate the apparent ileal digestibility of crude protein [24]. In order to avoid confounding effects from specific fatty acids contained in the HI larvae meal, the levels of the major fatty acids of the diets were adjusted by addition of individual amounts of soybean oil, palm oil and coconut fat to the diets. The rats had free access to the experimental diets. Water was constantly available *ad libitum* from nipple drinkers. The duration of the feeding experiment was 4 weeks.

### 2.2. Analysis of Feed Composition

The determination of DM and gross energy content, concentrations of crude nutrients [crude protein, crude fiber, crude ash, ether extract] and amino acids in the diets and the HI larvae meal was performed as described recently [9]. Total lipid fatty acid analysis of the HI larvae meal, the experimental fats and the diets was carried out as described previously in detail [25]. Total lipids from HI larvae meal and the experimental diets were extracted according to [26]. The concentration of chitin in the HI larvae meal was quantified using a modified method for glucosamine determination according to [27] as described in more detail [9].

### 2.3. Sample Collection

Rats were decapitated under CO_2_ anesthesia. Blood was collected in heparin-coated polyethylene tubes (AppliChem, Darmstadt, Germany). Subsequently, the plasma was separated from the remaining blood components by centrifugation (1100× *g*, 10 min) at 4 °C. The liver was removed, washed in ice cold NaCl solution (0.9%), weighed, and several small aliquots were collected. In addition, the right leg was removed and *M. gastrocnemius* and *M. soleus* were prepared and weighed. Immediately after collection, all tissue samples were snap-frozen in liquid nitrogen and stored at −80 °C until analysis.

### 2.4. Determination of Triglyceride and Cholesterol Concentrations in Liver and Plasma

Concentrations of triglyceride and cholesterol in liver and plasma were determined using enzymatic reagent kits as described recently [9]. Prior to measurement in the liver, total lipids were extracted from liver samples [26]. Subsequently, extracted lipids were dried and lipids were dissolved with chloroform and Triton X-100 (1:1, *v*/*v*) [28].

### 2.5. Determination of the Fatty Acid Composition of Hepatic Total Lipids

Fatty acid concentrations of hepatic total lipids were determined as fatty acid methyl esters by gas chromatrography-flame ionisation detection (GC-FID) as described previously in detail [25].

### 2.6. Histological Analysis of Liver

Liver lipid accumulation was evaluated by Oil Red O-staining and Hematoxylin and Eosin-staining of cryosectioned liver slices as described [9]. Stained sections were photographed with an EVOS M5000 microscope (Thermo Fisher Scientific, Dreieich, Germany).

### 2.7. Hepatic Activities of Lipogenic Enzymes

The activities of glucose-6 phosphate dehydrogenase (G6PD, EC 1.1.1.49) and malic enzyme (ME, EC 1.1.1.40) were determined in liver cytosolic fractions as described [29]. In brief, G6PD activity was measured using an assay from Sigma-Aldrich (Taufkirchen, Germany; cat. no. MAK015). ME activity was determined by incubation of the cytosolic fraction with malate and NADP^+^, monitoring the reduction of NADP^+^ spectrophotometrically, and normalizing to total cytosolic protein, according to [30]. Protein concentration of the cytosolic fractions was determined by an assay kit (BCA Protein assay, Interchim, Montluçon, France) with bovine serum albumin as standard.

### 2.8. Total RNA Extraction and qPCR Analysis

Frozen liver aliquots (15–20 mg) were used for total RNA extraction using TRIzol reagent (Invitrogen, Karlsruhe, Germany). Subsequently, total RNA was analyzed for quantity and quality using an Infinite 200 M microplate reader equipped with a NanoQuant plate (both from Tecan, Mainz, Germany). The average RNA concentration and A260/A280 ratio of all total RNA samples were 0.54 ± 0.16 µg/µL (n = 40) and 1.90 ± 0.03 (n = 40), respectively. The cDNA was synthesized as previously described [31]. The qPCR analysis was performed with a Rotor-Gene Q system (Qiagen, Hilden, Germany) using gene-specific primer pairs (Eurofins MWG Operon, Ebersberg, Germany) as recently described [32]. The properties of primers are presented in Supplemental Table S1. The qPCR data were normalized using the three most stable (*Canx*, *Mdh1*, *Sdha*) out of seven potential reference genes tested according to [33].

### 2.9. Statistical Analysis

Statistical analysis was performed using SPSS 27 statistical software (IBM, Armonk, NY, USA). The individual animal served as the experimental unit for all data except for daily feed intake and feed conversion ratio, for which the cage served as the experimental unit. All data were tested for normal distribution using the Shapiro–Wilk test and for variance homogeneity using Levene´s test. When the data showed normal distribution only after log transformation, the log-transformed data were used for statistical analysis. For statistical comparison of the lean (L-C) and obese control (O-C) groups, a Student´s *t*-test was performed when the data were normally distributed and homoscedastic. Normally distributed but heteroscedastic data were analyzed with a Welch´s *t*-test and data that did not follow a normal distribution were analyzed with a Mann–Whitney U test. The effect of protein replacement between the three obese groups (O-C, O-HI25, O-HI50) was analyzed using one-way analysis of variance (ANOVA) followed by a Tukey post-hoc test when the data were normally distributed and variances were homogeneous. The three obese groups were analyzed using Welch´s ANOVA in conjunction with the Games–Howell post-hoc test, if the data showed heterogeneity of variance. When there was no normal distribution, a one-way Kruskal–Wallis ANOVA was performed using the Mann–Whitney U test with Bonferroni correction as a post-hoc test. A *p*-value < 0.05 was regarded as statistically significant.

## 3. Results

### 3.1. Characterization of the Experimental Diets

Since casein was present in all three diets, the protein concentration of the diets was calculated using the standard *N*-to-protein conversion factor of 6.25, despite being aware that the protein concentration in the two diets containing HI larvae meal was overestimated due to the presence of chitin. Accordingly, the protein concentration in the diets increased in the order: C < HI25 < HI50 (Table 3). In contrast, the chitin-corrected protein concentration of the diets was similar. In addition, the sum of total amino acids in the diets was similar among the three diets. In addition, the gross energy content was similar between the diets. Due to decreasing amounts of cellulose with increasing replacement of casein by HI larvae meal, the concentration of crude fiber in the diets decreased in the following order: C > HI25 > HI50. The concentration of ether extract was slightly higher in diet HI50 than in diet HI25 and in diet HI25 than in diet C. The concentration of crude ash in the diets decreased in the following order: HI50 > HI25 > C. The levels of the dominating fatty acids [lauric acid (C12:0), α-linoleic acid (C18:2 n-6), oleic acid (18:1 n-9), palmitic acid (C16:0), myristic acid (C14:0), stearic acid (C18:0) and α-linolenic acid (C18:3 n-3)]—which contributed to > 91% of total fatty acids—were similar in the three diets due to the adjustment of fatty acid composition by using individual amounts of different dietary fats (Table 3).

### 3.2. Growth Performance and Organ Weights of the Rats

While body weights at weeks 0 and 1 did not differ between group L-C and group O-C, body weights at weeks 2, 3 and 4 were higher in group O-C than in group L-C (*p* < 0.05). Body weights did not differ across the obese groups at all time points (Table 4). Daily body weight gain and daily feed intake during weeks 1, 2, 3, 4 and 1–4 were higher in group O-C than in group L-C (*p* < 0.05), but daily body weight gain and daily feed intake during most weeks and weeks 1–4 did not differ across the obese groups. Only during week 1 daily body weight gain was higher in group O-HI25 than in group O-C (*p* < 0.05), but was not different between group O-HI50 and group O-C. Daily feed intake during week 4 was higher in group O-HI50 than in groups O-C and O-HI25 (*p* < 0.05). The feed conversion ratio during weeks 1, 2, 3 and weeks 1–4 was lower in group O-C than in group L-C (*p* < 0.05), but was not different across the obese groups. Absolute and relative liver weights of the rats were higher in group O-C than in group L-C (*p* < 0.05) but did not differ across the obese groups (Table 4). The weights of *M. soleus* and *M. gastrocnemius* were higher in group L-C than in group O-C (*p* < 0.05) but were not different across the obese groups.

### 3.3. Liver Lipid Accumulation and Liver and Plasma Lipid Concentrations

**Figure 1:**
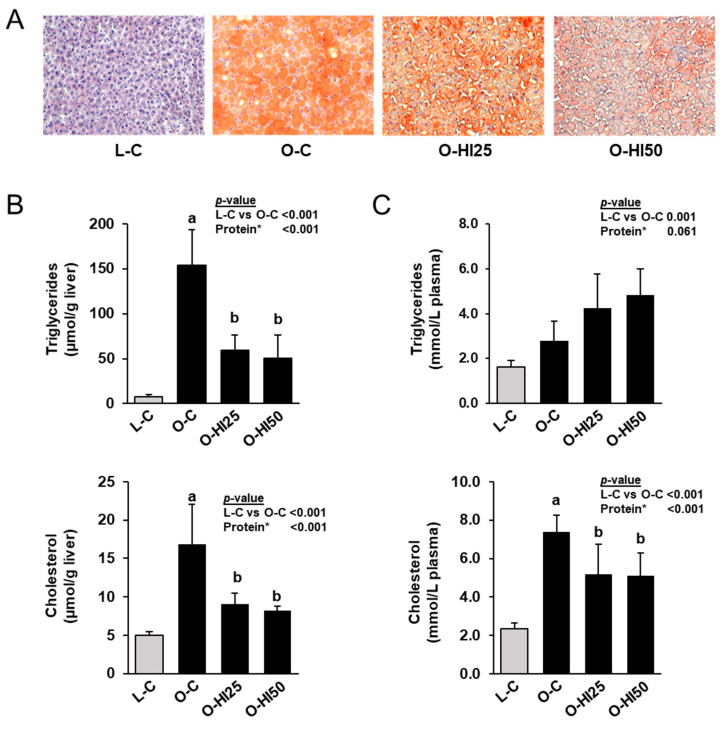
ORO-stained liver sections (**A**), triglyceride and cholesterol concentrations in liver (**B**) and plasma (**C**) of the rats. (**A**) Images are shown for one animal per group. (**B**,**C**) Bars represent means ± SD for n = 10 rats/group. * Effect of the protein source (C vs. HI25 vs. HI50) within the obese groups: ^a,b^ Bars without a common letter differ across the obese groups, *p* < 0.05.

ORO- and H&E-staining of liver sections revealed a healthy parenchyma structure with normal liver cell morphology, clearly visible nuclei and no lipid accumulation in group L-C (Figure 1A). In contrast, the liver parenchyma structure appeared pathological with enlarged liver cells, almost no visible nuclei and strong lipid accumulation in all obese groups. However, lipid accumulation of liver parenchyma was clearly less in groups O-HI50 and O-HI25 than in group O-C and less in group O-HI50 than in group O-HI25.

In line with the histological findings, the hepatic concentrations of triglycerides and cholesterol were markedly higher in group O-C than in group L-C, but lower in groups O-HI25 and O-HI50 than in group O-C (*p* < 0.05, Figure 1B). Plasma concentrations of triglycerides and cholesterol were also higher in group O-C than in group L-C (*p* < 0.05, Figure 1C). While plasma concentration of cholesterol was lower in groups O-HI25 and O-HI50 than in group O-C (*p* < 0.05), plasma concentration of triglycerides did not differ across the obese groups. Liver and plasma triglyceride and cholesterol concentrations did not differ between groups O-HI25 and O-HI50.

### 3.4. Hepatic Concentrations of Total Lipid Fatty Acids

Hepatic concentrations of most individual fatty acids (C12:0, C14:0, C14:1 n-5, C16:0, C16:1 n-7, C18:0, C18:1 n-9, C18:2 n-6, C18:3 n-3, C20:3 n-6), and the sum of all individual fatty acids were markedly higher in group O-C than in group L-C (*p* < 0.05, Table 5), with C18:1 n-9, C16:0 and C16:1 n-7 contributing to almost 90% of total hepatic fatty acids in group O-C. Hepatic concentrations of C14:0, C14:1 n-5, C16:0, C16:1 n-7, C18:1 n-9, C20:3 n-6, C20:4 n-6 and the sum of total fatty acids were lower in groups O-HI25 and O-HI50 than in group O-C (*p* < 0.05). In group O-HI50, hepatic concentrations of C14:1 n-5, C16:0, C16:1 n-7, C18:0, C18:1 n-9, C20:4 n-6 and the sum of total fatty acids was lower than in group O-HI25 (*p* < 0.05).

The calculated Δ5- and Δ6-desaturation indices were lower in group O-C than group L-C, whereas the calculated Δ9-desaturation index was markedly higher in group O-C than in group L-C (*p* < 0.05). While ∆5- and ∆9-desaturation indices were markedly lower in groups O-HI25 and O-HI50 than in group O-C, the ∆6-desaturation index was only lower in group O-HI50 than in group O-C (*p* < 0.05).

### 3.5. Expression and Activity of Enzymes Involved in Lipid Synthesis and Bile Acid Synthesis in the Liver

Hepatic mRNA levels of most genes involved in fatty acid synthesis (*Acly*, *Acaca*, *Fasn*, *G6pd*, *Me1*), desaturation of fatty acids (*Fads1*, *Fads2*, *Scd1*) and triglyceride synthesis (*Gpam*) were higher in group O-C than in group L-C (*p* < 0.05, Table 6), whereas the mRNA levels of all these genes, except *Acaca*, were lower in groups O-HI25 and O-HI50 than in group O-C (*p* < 0.05). The mRNA level of *Acaca* tended to be lower in groups O-HI25 and O-HI50 than in group O-C (*p* < 0.1). While the mRNA levels of most of these genes did not differ between groups O-HI50 and O-HI25, the mRNA levels of *Fads1*, *Fads2* and *G6pd* were lower in group O-HI50 than in group O-HI25 (*p* < 0.05). The mRNA levels of *Ldlr* and *Hmgcr* were higher (*p* < 0.05) and tended to be higher (*p* < 0.1), respectively, in group O-C than in group L-C. The mRNA level of *Hmgcr* was lower in groups O-HI25 and O-HI50 than in group O-C (*p* < 0.05), whereas the mRNA level of *Ldlr* was not different across the obese groups. The mRNA level of the rate-limiting enzyme involved in bile acid synthesis, *Cyp7A1*, was not different across all groups.

**Figure 2:**
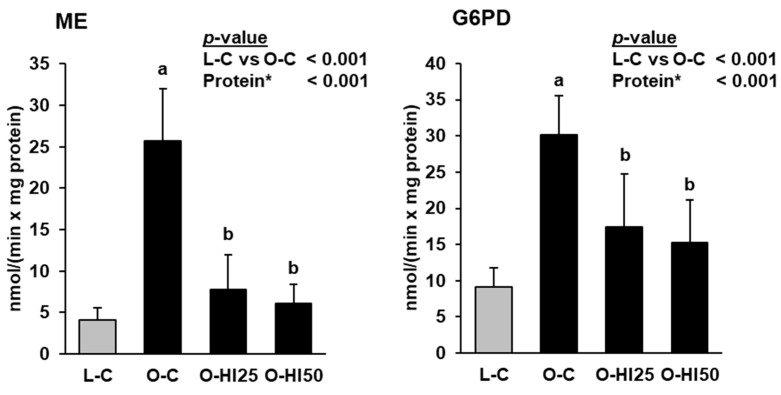
Hepatic activities of malic enzyme (ME) and glucose-6-phosphate dehydrogenase in the rats. Bars represent means ± SD for n = 10 rats/group. * Effect of the protein source (C vs. HI25 vs. HI50) within the obese groups: ^a,b^ Bars without a common letter differ across the obese groups, *p* < 0.05.

Hepatic activities of ME and G6PD were markedly higher in group O-C than in group L-C, but lower in groups O-HI25 and O-HI50 than in group O-C (*p* < 0.05, Figure 2).

## 4. Discussion

In the present study, the hypothesis was tested that dietary HI larvae meal attenuates the development of liver steatosis and hyperlipidemia in the obese Zucker rat. In order to investigate this hypothesis, the protein from casein was partially replaced with protein from HI larvae meal at two different replacement levels (25% and 50%). Replacement of protein from casein with HI larvae meal at higher levels was not possible, because the protein content of the HI larvae meal was markedly lower than that of casein. Thus, only the effect of partial but not complete replacement of casein by HI larvae meal could be investigated. Although the protein concentration of the diets calculated using the standard *N*-to-protein conversion factor of 6.25 indicated that replacement of casein with HI larvae meal in the diets was not isoproteinogenous, it has to be considered that the protein concentration in the two HI larvae meal-containing diets was overestimated due to the presence of the nonprotein *N*-compound chitin. This was obvious when the calculated protein concentration in diets HI25 and HI50 was corrected for the amount of chitin present in the HI larvae meal. In line with this, calculating the sum of total dietary amino acids from the analyzed amounts of all individual amino acids revealed that the dietary protein concentration was comparable across the three diets. Considering this, partial replacement of casein by HI larvae meal was isoproteinogenous. In addition, the amount of fat and the fatty acid composition of the dietary fat was adjusted across the diets by using mixtures of different fats. Owing to this, the biological effects induced by replacing casein with HI larvae meal cannot be ascribed to specific fatty acids of the HI larvae meal.

The results of this study convincingly demonstrate that liver triglyceride and cholesterol concentrations, plasma cholesterol concentration and hepatic mRNA levels and/or activities of cholesterogenic and lipogenic genes—which are mainly regulated at the transcriptional level *via* sterol regulatory element-binding proteins [34]—were markedly reduced in rats fed HI larvae meal. In addition, our study showed that hepatic concentrations of fatty acids originating from *de novo*-lipogenesis, such as C16:0, C16:1 n-7 and C18:1 n-9, were strongly reduced in both groups of rats fed HI larvae meal. Since these fatty acids contributed to almost 90% of total hepatic fatty acids, the sum of all individual fatty acids in the liver were also reduced in rats fed HI larvae meal. Moreover, the calculation of fatty acid desaturation indices from hepatic fatty acid concentrations revealed that ∆9, ∆6 and ∆5 desaturation pathways, which are responsible for the synthesis of monounsaturated (∆9) and polyunsaturated (∆6, ∆5) fatty acids, were clearly lowered in rats fed HI larvae meal. Our observation that the mRNA level of *Cyp7a1* did not differ across the obese groups of rats indicates that an increased hepatic bile acid synthesis from cholesterol did not contribute to the lowering of hepatic cholesterol concentration in rats fed the HI larvae meal. According to these results, the hypothesis of our study could be clearly verified that HI larvae meal, like TM larvae meal [8,9,10,11], attenuates liver steatosis and dyslipidemia in obese Zucker rats. In addition, the observations from our study indicate that the lipid-lowering effect of HI larvae meal is mainly caused by a profound inhibition of hepatic fatty acid, triglyceride and cholesterol synthesis. Although the liver lipid concentrations were not statistically different between the two groups of rats fed HI larvae meal, the observation that liver lipid concentrations were numerically lower and hepatic concentrations of C16:0, C16:1 n-7 and total fatty acids and hepatic mRNA levels of several lipogenic genes (*Fads1*, *Fads2*, *G6pd*) were significantly lower in group O-HI50 than in group O-HI25, respectively, suggests that the inhibitory effect of HI larvae meal on lipid synthesis is dose-dependent. We are confident that all these findings are indicative of a marked inhibition of hepatic lipid synthesis by HI larvae meal, but not of a reduced bioavailability of fatty acids from the HI larvae meal-containing diets. In the latter case, body weight gain would have been reduced due to a reduced intake of digestible energy. This, however, was not the case. In addition, in a recent study with obese Zucker rats fed a diet with TM larvae meal, the apparent total tract digestibility of ether extract was reported to be 96% [9]. This indicates that the digestibility of fatty acids from insect meal in rats is very high and a reduced bioavailability of fatty acids from HI larvae meal is likely not causative. Moreover, the main part of dietary fat in diet HI50 was derived from other fat sources, such as soybean oil and coconut fat, which are also highly digestible in rats. Furthermore, the marked repression of lipogenic genes in the liver, the strong inhibition of lipogenic enzyme activities and the strong reduction of fatty acids in the liver derived from *de novo*-fatty acid synthesis in group O-HI50 does not support the assumption that an enhanced lipid mobilization from the liver was the main reason for the strong antisteatotic effect of HI larvae meal. However, future studies are warranted to clarify this issue. In the present study, adipose tissues weights were not weighed or histopathologically examined. However, the unaltered body weights, organ weights and feed intake across the obese groups did not suggest that adipose tissue weights or lipid deposition in adipose tissue were affected by casein replacement with HI larvae meal.

Regarding the heterogenous composition of the HI larvae meal, various bioactive compounds might be responsible for its lipid-lowering activity. One possible candidate is chitin, a structural polysaccharide consisting of β-(1–4)-*N*-acetyl-D-glucosamine monomers which largely serves as a fermentation substrate in the large intestine of monogastric animals. According to the chitin content analyzed in the HI larvae meal (13% of FM), the diets HI25 and HI50 contained approximately 1.5% and 3.0% chitin, respectively. Several reports exist in the literature showing that feeding chitosan—the deacetylation product of chitin—and chitosan oligosaccharides at comparable chitin levels as in our study exerts potent antisteatotic and lipid-lowering effects in different rat models of fatty liver and obesity [35,36,37,38]. According to these studies, different mechanisms including the inhibition of hepatic lipid synthesis [39] and a reduction of systemic and hepatic inflammation [38,40], which is known to promote hepatic lipid synthesis through stimulating NF-κB signaling pathway [41], have been identified as important antisteatotic effects of chitosan. In a recent study with obese Zucker rats, feeding diets enriched with chitin *via* supplementation of insects´ cuticles was also found to cause a pronounced inhibition of hepatic lipid accumulation and hepatic lipid concentrations [42]. In addition, this study showed that feeding the chitin-containing diets modified the gut microbiota community structure in a favorable manner with increased abundances of bacterial families [42], which are known to strengthen the gut barrier, decrease systemic and hepatic inflammation and attenuate hepatic steatosis in various rodent models of obesity, fatty liver and metabolic syndrome [43,44,45,46,47]. Although we did not analyze the gut microbiota structure of the rats, it is not unlikely that feeding of the HI larvae meal-diets has also modified the gut microbiota composition of the obese rats in a beneficial manner, thereby contributing to the strong antisteatotic effects observed. In line with this, several studies with broilers and pigs have demonstrated that feeding of different insect larvae meals affects the gut microbiota composition [12,48,49]. However, in order to clarify this speculative issue, future studies have to demonstrate that HI larvae meal alters the gut microbiota composition in a favorable manner in the obese Zucker rat. In addition, future studies have to demonstrate that the chronic systemic inflammatory condition and the impaired liver function, which is known to develop in obese Zucker rats [42], is attenuated by partial replacement of casein by HI larvae meal. Moreover, considering recent indications that casein promotes pro-inflammatory activities, disturbs liver lipid metabolism and induces liver steatosis in obese Zucker rats when compared to soy protein isolate [50,51], it has to be clarified whether the markedly higher liver lipid accumulation in group O-C compared to groups O-HI25 and O-HI50 was caused by either a pro-inflammatory effect of casein or an anti-inflammatory effect of HI larvae meal. However, considering the relative liver weight and the body weight gain throughout the experiment, both of which did not differ across the obese groups of rats, no evidence for a negative effect of casein compared to HI larvae meal has been gained in the present study.

Apart from chitin, the protein fraction from HI larvae meal might be also a source of bioactive compounds with lipid-lowering activities. Indeed, cholesterol-lowering peptide sequences, which were identified in soybean protein [16], have been made responsible for the cholesterol-lowering activities of different dietary proteins, such as soybean and other legume proteins [52,53,54]. Despite the current lack of evidence for the presence of bioactive peptides with lipid-lowering activities in HI larvae protein, the occurrence of bioactive peptides with other biological activities, such as ACE-inhibitory peptides, has been reported for insect protein in several studies [19,20]. Based on this, it appears not unlikely that peptides with lipid-lowering actions are also present in HI larvae proteins. Future studies are required to address this issue. A further fraction of the HI larvae meal that was present in significant amounts is fat, despite the HI larvae meal having been partially defatted by the producer. However, we exclude the possibility that specific fatty acids in the HI larvae meal are responsible for its lipid-lowering activity, because both the amount of fat and the fatty acid composition of the dietary fat were adjusted across the diets by using mixtures of different fats.

A further factor that could account for the biological activity of HI larvae meal is the amino acid composition. Regarding TM larvae meal, one characteristic feature of its amino acid composition is a low concentration of methionine when compared to casein. This is relevant with regard to the lipid-lowering activity of TM larvae meal, because methionine restriction was reported to cause a marked inhibition of hepatic *de novo*-lipogenesis and cholesterogenesis and the attenuation of liver steatosis [55,56,57,58,59]. Based on this, we have recently raised the hypothesis that the lipid-lowering effect of isoproteinogenic replacement of casein by TM larvae meal is due to methionine restriction [9]. However, in a subsequent study, in which the TM meal-containing diet was supplemented with methionine to a similar level as in the casein diet, the lipid-lowering effect of TM larvae meal was still observed [10]. In addition, an increased supply of cysteine to the TM larvae meal-containing diet or the adjustment of essential amino acid levels between the TM larvae meal diet and the casein diet did not reverse the lipid-lowering effect of TM larvae meal [10]. This clearly indicated that a divergent amino acid composition between TM larvae meal and casein, a low methionine level in TM larvae meal and a decreased cysteine synthesis as a consequence of a reduced methionine availability resulting from feeding TM larvae meal are not responsible for the lipid-lowering action of TM larvae meal. In the present study, the concentration of methionine in diets HI25 and HI50 was 10 and 20%, respectively—lower than in diet C (5.3 g/kg diet)—but the dietary methionine concentration in diets HI25 (4.8 g/kg diet) and HI50 (4.3 g/kg diet) was clearly higher than the maintenance requirement of methionine for rats (2.3 g methionine + cysteine/kg diet, from which cysteine may supply up to 50%; [22]). Thus, we propose that differences in the dietary concentration of methionine between groups are not causative for the lipid-lowering effect of HI larvae meal.

## 5. Conclusions

The present study shows that HI larvae meal, like TM larvae meal, attenuates liver steatosis and dyslipidemia in obese Zucker rats. This suggests that HI larvae meal serves not only as an alternative source of dietary protein, which can be sustainably produced using regionally available agro-industrial side-streams, but also as a functional food protecting from obesity-induced metabolic disorders such as fatty liver and dyslipidemia.

## Figures and Tables

**Table 1:** Composition of the experimental diets.

	C	HI25	HI50
*Components, g/kg*			
Cornstarch	473.5	473.5	473.5
Casein	200	150	100
HI larvae meal	-	115.9	231.8
Sucrose	100	100	100
Mineral mix ^1^	35	35	35
Vitamin mix ^2^	10	10	10
Soybean oil	29.2	27.1	25.1
Palm oil	28.4	14.2	-
Coconut fat	7.4	11	14.5
Cellulose	110	56.8	3.65
L-cysteine	1.5	1.5	1.5
TiO_2_	5	5	5

^1,2^ The mineral and vitamin mix were identical as in our recent publication [9].

**Table 2:** Analyzed concentrations of crude nutrients, chitin and fatty acids of the HI larvae meal.

	HI Larvae Meal
*Crude nutrients and chitin*	
Dry matter (DM), % fresh matter	92.3
Protein (*N* × 4.67), % DM	43.1
Ether extract, % DM	11.9
Crude ash, % DM	7.65
Crude fiber, % DM	14.6
Chitin, % DM	14.1
*Fatty acids, g/100 g total fatty acids*	
C10:0	1.09
C12:0	53.7
C14:0	8.34
C16:0	11.7
C16:1 n-7	2.88
C18:0	1.68
C18:1 n-9	8.33
C18:2 n-6	11.3
C18:3 n-3	0.89

**Table 3:** Concentrations of crude nutrients and energy, total lipid fatty acid composition and amino acid concentrations of the experimental diets.

	C	HI25	HI50
*Analyzed concentrations of crude nutrients and energy*			
Dry matter (DM), % fresh matter	89.1	88.7	89
Protein (*N* × 6.25), % DM	20.1	21.2	23.3
Ether extract, % DM	6.8	7.6	8.0
Crude ash, % DM	3.1	4.0	4.7
Crude fiber, % DM	8.1	6.1	4.3
Gross energy, MJ/kg DM	20.9	20.9	20.7
*Calculated chitin-corrected protein concentration*			
Protein_Chitin-corr._ [(total *N*-chitin-*N*) × 6.25], % DM	20.1	19.9	20.6
Fatty acid composition, g/100 g total fatty acids *			
C8:0	3.86	1.87	0.13
C10:0	3.08	1.69	0.52
C12:0	22.9	24.2	25.4
C14:0	8.52	6.29	4.34
C16:0	13.1	15.4	16.9
C16:1 n-7	0.10	0.74	1.33
C18:0	3.34	3.06	2.78
C18:1 n-9	18.1	19.4	20.2
C18:2 n-6	23.0	22.9	23.7
C18:3 n-6	0.20	0.25	0.39
C18:3 n-3	2.18	2.00	2.00
C20:0	0.68	0.71	0.71
C20:1 n-9	0.16	0.16	0.15
C20:4 n-6	0.24	0.30	0.39
C22:0	0.23	0.55	0.47
C22:1 n-9	0.34	0.36	0.43
*Amino acids, g/kg FM*			
Alanine	6.0	10.1	14.3
Arginine	6.3	8.5	10.7
Aspartic acid	13.5	14.8	16.0
Cysteine	1.6	1.7	1.8
Glutamic acid	42.8	38.2	33.6
Glycine	3.4	5.6	7.8
Histidine	5.4	6.1	6.8
Isoleucine	8.9	9.0	9.2
Leucine	17.1	16.6	16.2
Lysine	14.7	14.1	13.5
Methionine	5.3	4.8	4.3
Phenylalanine	9.6	9.3	9.0
Proline	20.7	18.8	16.8
Serine	11.2	10.8	10.3
Threonine	8.0	8.1	8.1
Tryptophan	2.0	2.9	3.9
Tyrosine	9.6	10.1	10.6
Valine	11.9	12.1	12.2
Sum of total amino acids	198	202	205

* Only fatty acids with concentrations > 0.1 g/100 g total fatty acids are shown.

**Table 4:** Performance and organ weights of lean rats fed a control diet with casein (L-C) and obese rats fed either the control diet with casein (O-C) or the control diet in which 25% (O-HI25) and 50% (O-HI50) of the protein from casein was replaced with protein from HI larvae meal for 4 weeks.

Group	L-C	O-C	O-HI25	O-HI50	*p*-Value	
					L vs. O	Protein Source *
Body weight, g						
week 0	257 ± 19	260 ± 32	256 ± 33	264 ± 28	0.799	0.847
week 1	294 ± 23	316 ± 31	321 ± 31	324 ± 22	0.092	0.780
week 2	322 ± 24	379 ± 33	380 ± 31	386 ± 19	<0.001	0.842
week 3	343 ± 25	427 ± 34	426 ± 30	428 ± 16	<0.001	0.985
week 4	364 ± 27	462 ± 32	460 ± 33	460 ± 14	<0.001	0.985
Daily body weight gain, g/d						
week 1	5.26 ± 0.73	7.90 ± 1.19 ^b^	9.32 ± 1.15 ^a^	8.64 ± 2.21 ^ab^	<0.001	0.042
week 2	3.95 ± 0.83	8.94 ± 1.03	8.44 ± 1.35	8.74 ± 0.82	<0.001	0.537
week 3	2.96 ± 0.90	6.96 ± 0.87	6.56 ± 1.25	6.11 ± 1.00	<0.001	0.208
week 4	3.04 ± 0.63	4.95 ± 0.91	4.86 ± 1.13	4.52 ± 0.78	<0.001	0.578
week 1 to 4	3.80 ± 0.52	7.20 ± 0.78	7.29 ± 1.05	7.00 ± 0.87	<0.001	0.766
Daily feed intake, g/d						
week 1	24.9 ± 1.6	31.9 ± 1.9	32.6 ± 3.2	33.2 ± 2.4	<0.001	0.718
week 2	22.6 ± 1.2	34.3 ± 1.5	33.9 ± 2.3	36.5 ± 2.6	<0.001	0.177
week 3	21.3 ± 1.0	33.2 ± 1.5	33.2 ± 2.6	36.1 ± 2.7	<0.001	0.116
week 4	21.5 ± 1.2	30.3 ± 1.4 ^b^	29.5 ± 1.1 ^b^	33.3 ± 2.1 ^a^	<0.001	0.007
week 1 to 4	22.6 ± 1.1	32.4 ± 1.4	32.3 ± 2.0	34.8 ± 2.4	<0.001	0.131
Feed conversion ratio, g/g						
week 1	4.78 ± 0.43	4.06 ± 0.43 ^b^	3.52 ± 0.51 ^a^	3.91 ± 0.71 ^ab^	0.028	0.333
week 2	5.79 ± 0.76	3.82 ± 0.20	4.06 ± 0.54	4.21 ± 0.54	0.001	0.432
week 3	7.42 ± 1.34	4.78 ± 0.19	5.21 ± 1.18	6.02 ± 1.05	0.002	0.136
week 4	7.24 ± 1.17	6.23 ± 0.89	6.21 ± 1.10	7.58 ± 1.63	0.161	0.181
week 1 to 4	5.96 ± 0.37	4.52 ± 0.33	4.48 ± 0.65	5.04 ± 0.81	0.001	0.329
Organ weights						
Liver, g	14.0 ± 1.4	31.9 ± 6.6	28.1 ± 5.2	30.2 ± 2.3	<0.001	0.372
Liver, % of BW	3.85 ± 0.19	6.85 ± 1.12	6.11 ± 1.08	6.56 ± 0.53	<0.001	0.222
*M. soleus*, g	0.13 ± 0.01	0.11 ± 0.01	0.10 ± 0.01	0.10 ± 0.01	<0.001	0.884
*M. gastrocnemius, g*	2.01 ± 0.16	1.40 ± 0.10	1.39 ± 0.15	1.38 ± 0.09	<0.001	0.966

Data are means ± SD for n = 10 rats/group (body weight, body weight gain, organ weights) and n = 5 rats/group (feed intake, feed conversion ratio). * Effect of the protein source (C vs. HI25 vs. HI50) within the obese groups: ^a,b^ Bars without a common letter differ across the obese groups, *p* < 0.05.

**Table 5:** Hepatic concentrations of total lipid fatty acids of lean rats fed a control diet with casein (L-C) and obese rats fed either the control diet with casein (O-C) or the control diet in which 25% (O-HI25) and 50% (O-HI50) of the protein from casein was replaced with protein from HI larvae meal for 4 weeks.

Group	L-C	O-C	O-HI25	O-HI50	*p*-Value	
					L vs. O	Protein Source *
Fatty acid, µmol/g liver						
C12:0	0.54 ± 0.10	1.76 ± 2.11	1.54 ± 0.55	1.54 ± 0.26	0.002	0.224
C14:0	0.98 ± 0.12	8.54 ± 3.01 ^a^	6.41 ± 1.77 ^ab^	5.26 ± 0.74 ^b^	<0.001	0.005
C14:1 n-5	n.d.	1.92 ± 1.39 ^a^	0.95 ± 0.17 ^b^	0.66 ± 0.16 ^c^	-	<0.001
C16:0	15.8 ± 1.3	140 ± 56 ^a^	80.2 ± 20.7 ^b^	61.7 ± 9.2 ^c^	<0.001	<0.001
C16:1 n-7	1.90 ± 0.45	54.2 ± 26.0 ^a^	24.0 ± 6.1 ^b^	17.1 ± 3.4 ^c^	<0.001	<0.001
C18:0	9.55 ± 0.72	12.7 ± 4.8 ^a^	12.4 ± 2.3 ^a^	10.0 ± 0.94 ^b^	0.004	0.017
C18:1 n-9	8.25 ± 1.47	151 ± 79 ^a^	71.0 ± 23.2 ^b^	55.2 ± 8.4 ^b^	<0.001	<0.001
C18:2 n-6	7.22 ± 0.93	11.0 ± 5.1	12.0 ± 3.9	13.6 ± 3.7	0.049	0.225
C18:3 n-3	0.17 ± 0.03	0.78 ± 0.36	0.79 ± 0.48	0.85 ± 0.27	<0.001	0.705
C20:3 n-6	0.33 ± 0.06	1.27 ± 2.71 ^a^	1.17 ± 0.24 ^b^	1.27 ± 0.17 ^b^	0.096	0.001
C20:4 n-6	9.49 ± 1.43	9.31 ± 1.34 ^a^	8.71 ± 1.44 ^a^	7.14 ± 0.74 ^b^	0.775	0.001
C22:5 n-3	0.23 ± 0.05	0.24 ± 0.14 ^b^	0.56 ± 0.10 ^a^	0.55 ± 0.15 ^a^	0.866	<0.001
C22:6 n-6	1.83 ± 0.44	1.83 ± 0.34 ^b^	2.23 ± 0.42 ^a^	1.85 ± 0.30 ^ab^	0.984	0.030
Sum	56.5 ± 5.5	396 ± 176 ^a^	223 ± 58 ^b^	178 ± 22 ^c^	<0.001	<0.001
Desaturation indices						
C20:4 n-6/C20:3 n-6 (Δ5)	28.9 ± 3.6	21.6 ± 9.4 ^a^	7.55 ± 1.03 ^b^	5.66 ± 0.49 ^c^	0.034	<0.001
C20:4 n-6/C18:2 n-6 (Δ6)	1.32 ± 0.15	0.98 ± 0.36 ^a^	0.77 ± 0.19 ^a^	0.56 ± 0.15 ^b^	0.017	0.004
C18:1 n-9/C18:0 (Δ9)	0.86 ± 0.15	19.3 ± 30.5 ^a^	5.75 ± 1.50 ^b^	5.51 ± 0.72 ^b^	<0.001	0.001

Data are means ± SD for n = 10 rats/group. * Effect of the protein source (C vs. HI25 vs. HI50) within the obese groups: ^a,b,c^ Bars without a common letter differ across the obese groups, *p* < 0.05. n.d., not detected.

**Table 6:** Hepatic mRNA levels of genes involved in lipid metabolism of lean rats fed a control diet with casein (L-C) and obese rats fed either the control diet with casein (O-C) or the control diet in which 25% (O-HI25) and 50% (O-HI50) of the protein from casein was replaced with protein from HI larvae meal for 4 weeks.

Group	L-C	O-C	O-HI25	O-HI50	*p*-Value	
					L vs. O	Protein Source *
*Fatty acid synthesis*						
*Acly*	0.33 ± 0.16	1.00 ± 0.33 ^a^	0.70 ± 0.23 ^ab^	0.48 ± 0.27 ^b^	<0.001	0.002
*Acaca*	0.36 ± 0.20	1.00 ± 0.27	0.74 ± 0.35	0.66 ± 0.33	<0.001	0.055
*Fasn*	0.33 ± 0.15	1.00 ± 0.39 ^a^	0.90 ± 0.36 ^ab^	0.57 ± 0.27 ^b^	<0.001	0.035
*G6pd*	0.28 ± 0.08	1.00 ± 0.39 ^a^	0.56 ± 0.35 ^b^	0.27 ± 0.18 ^c^	<0.001	<0.001
*Me1*	0.27 ± 0.15	1.00 ± 0.39 ^a^	0.45 ± 0.18 ^b^	0.30 ± 0.07 ^b^	<0.001	<0.001
*Desaturation of fatty acids*						
*Fads1*	0.69 ± 0.25	1.00 ± 0.36 ^a^	0.44 ± 0.16 ^b^	0.26 ± 0.10 ^c^	<0.001	<0.001
*Fads2*	0.38 ± 0.08	1.00 ± 0.25 ^a^	0.51 ± 0.17 ^b^	0.36 ± 0.12 ^c^	<0.001	<0.001
*Scd1*	0.43 ± 0.17	1.00 ± 0.24 ^a^	0.85 ± 0.35 ^ab^	0.60 ± 0.16 ^b^	<0.001	0.012
*Triglyceride synthesis*						
*Gpam*	0.15 ± 0.04	1.00 ± 0.74 ^a^	0.46 ± 0.33 ^b^	0.22 ± 0.10 ^b^	<0.001	0.001
*Cholesterol homeostasis*						
*Hmgcr*	0.69 ± 0.36	1.00 ± 0.36 ^a^	0.68 ± 0.33 ^b^	0.65 ± 0.22 ^b^	0.075	0.044
*Ldlr*	0.72 ± 0.23	1.00 ± 0.31	1.01 ± 0.30	0.91 ± 0.29	0.043	0.503
*Bile acid synthesis*						
*Cyp7a1*	1.12 ± 0.68	1.00 ± 0.51	0.63 ± 0.32	0.50 ± 0.23	0.682	0.049

Data are means ± SD for n = 10 rats/group. * Effect of the protein source (C vs. HI25 vs. HI50) within the obese groups: ^a,b,c^ Bars without a common letter differ across the obese groups, *p* < 0.05.

## Data Availability

All data are presented in the manuscript.

## Supplementary Materials

The following supporting information can be downloaded at: https://www.mdpi.com/article/10.3390/nu15020287/s1, Table S1: Characteristics of gene-specific primers used for qPCR analysis.
