# Supplementary material for: Feeding of Hermetia illucens Larvae Meal Attenuates Hepatic Lipid Synthesis and Fatty Liver Development in Obese Zucker Rats
Source: Nutrients. 2023 Jan 6;15(2):287. doi: 10.3390/nu15020287 (PMC9861802; doi:10.3390/nu15020287)
Supplement: Supplementary file 1 [file nutrients-15-00287-s001.zip › nutrients-2132018-supplementary.pdf]

Supplementary file 1

Table S1 **Characteristics of gene-specific primers used for qPCR analysis**

Gene symbol	Primer sequence	NCBI GenBank	Annealing T	Product size (bp)
Reference genes				
Canx	CCAGATGCAGATCTGAAGAC<br>CTGGGTCCTCAATTTCACGT	NM_172008	60	175
Mdh1	CAG ACA AAG AAG AGG TTG CC<br>CGT CAG GCA GTT TGT ATT GG	NM_033235.1	60	206
Sdha	GCC TCC GTG GTT GAG CTA GAA<br>CGA CAC AGC AAC ACC GAT GG	NM_130428.1	60	136
Target genes				
Acly	TCA GTC CCA AGT CCA AGA TCC C<br>ACG GGTAGA CCA TAG CAG CC	NM_016987	60	166
Acaca	CGTACGACGTTCGCCATAACC<br>GCTGGCGATGCTGTACACTG	NC_051345.1	60	237
Fads1	CAT TTT CCA GCA CCA CGC CAA<br>TAG AGA GGC AGC AAG GCT GG	NM_053445.2	60	188
Fads2	CAT CGA CCG CAA GGT CTA CAA C<br>CTT GCC CAC GAA ATC CAG GTC	NM_031344.2	60	139
Fasn	AGG TGC TAG AGG CCC TGC TA<br>GTG CAC AGA CAC CTT CCC AT	NM_017332.1	60	281
G6pd	TTG TAC CAG GGT GAT GCC TTC C<br>GCT CAC TCT GTT TGC GGA TGT C	NM_017006.2	60	199
Gpam	TGTACGCTGAGAGTGCCACA<br>AGGGAACCCTACAGCACCAC	NC_051336.1	60	190
Hmgcr	TGG CAG GAC GCA ACC TCT AC<br>GGC AGC AGG TTT CTT GCT GG	NM_013134.2	60	173
Ldlr	ACA GTG TCC TCC CAA GTC CAA<br>GCA AAT GTG GAT CTC GTC CTC	NM_175762.2	60	222
Me1	CTG CCT TGG GGA TTG CTC AT<br>GAG AGA AGC ACG CCC CTT A	NM_012600.3	60	128
Scd1	GTA CTA CAA GCC TGG CCT C<br>CAC CCA GGG AAA CCA GGA T	NM_139192.2	60	227
Cyp7a1	GCG GGA AAG CAA AGA CCA CC<br>ACT TGA CTT GGC TCT CCA CA	NM_012942.2	60	180
